# Chemokines as potential biomarkers for predicting the course of COVID-19 – a review of the literature

**DOI:** 10.3389/fimmu.2025.1662643

**Published:** 2025-09-23

**Authors:** Blanka Wolszczak-Biedrzycka, Beata Cieślikiewicz, Filip Studniarz, Łukasz Dąbrowski, Mateusz Fąs, Krystyna Matyszkiewicz–Suchodolska, Monika Harasimowicz, Justyna Dorf

**Affiliations:** ^1^ Department of Psychology and Sociology of Health and Public Health, University of Warmia and Mazury in Olsztyn, Olsztyn, Poland; ^2^ The Oncology Center of the Region of Warmia and Mazury in Olsztyn, Clinical Hospital of the Ministry of the Interior and Administration, Olsztyn, Poland; ^3^ Medical Laboratory, “Diagnostyka” Olsztyn, Olsztyn, Poland; ^4^ Department of Clinical Laboratory Diagnostics, Medical University of Bialystok, Bialystok, Poland

**Keywords:** COVID-19, cytokine storm, chemokines, SARS-CoV-2, biomarkers

## Abstract

Since the beginning of the COVID-19 pandemic, research has been ongoing to find the best diagnostic parameters to identify patients with a high risk of severe infection. Numerous studies have examined chemokine biomarkers in COVID-19 as a biomarker for high risk patients. The four main structural proteins of the SARS-CoV-2, spike protein, membrane protein, envelope protein and nucleocapsid protein enable the virus to penetrate host cells and stimulate the immune system. SARS-CoV-2 enters host cells via ACE2 in upper respiratory tract the virus entries by binding to the spike protein. Uncontrolled activation and enhancement of the immune response leads to massive release of cytokines and chemokines known as cytokine storm (CS). Chemokines are described as important cytokines in COVID-19 with a potential role as prognostic factor particularly for the severity of the infection and the risk of death from complications, to identify high-risk patients. Our review contains chemokines (CCL2, CCL3, CCL5, CXCL8, CXCL10), which level is significantly higher in patients with COVID-19 infection vs control individuals.

## Introduction

From the beginning of the pandemic until July 2024, more than 775 million people worldwide have been diagnosed with COVID-19, and the virus claimed 7 million lives. In Poland, there were 6,517,494 confirmed cases of COVID-19 and 119,622 fatalities (1). The high incidence of severe infections and the high fatality rate prompted research into the pathogenesis and significance of the cytokine storm in COVID-19.

Overproduction of pro-inflammatory cytokines and chemokines contributes to pneumonia and, in extreme cases, acute respiratory distress syndrome (ARDS) (2). Research has shown that the levels of pro- and anti-inflammatory cytokines and chemokines are correlated with the severity of SARS-CoV-2 infection and the risk of death from complications (3). Normal or reduced white blood counts (WBC) and lymphocytopenia are observed in most patients with mild and moderate SARS-CoV-2 infection. However, in patients with severe COVID-19, the levels of circulating neutrophils, plasma D-dimer levels and serum urea levels are elevated, whereas lymphocyte counts are reduced (4). These patients are also characterized by higher levels of cytokines and chemokines, mainly interleukin 6 (IL-6), interleukin 10 (IL- 10), and tumor necrosis factor alpha (TNF-α) (5, 6). An increase in the levels of interleukin 2 (IL-2), interleukin 7 (IL-7), macrophage colony-stimulating factor (M-CSF), granulocyte colony-stimulating factor (G -CSF), granulocyte-macrophage colony-stimulating factor (GM- CSF), interferon gamma-induced protein 10 (IP-10), monocyte chemoattractant protein-1 (MCP-1), and macrophage inflammatory protein-1 alpha (MIP 1-α) has also been observed in patients with severe symptoms of infection who require hospitalization in an intensive care unit (ICU) (7–11). Last researches shown that the binding of CCR1 and CXCR6 to monocytes and CD8 T lymphocytes influence on the course of COVID-19. An elevated expression of genetics-risk genes, increase in inflammatory cytokines and chemokines (12–15).

Many COVID-19 patients still require hospitalization and die from complications, which is why new biomarkers that are potentially useful for diagnosing the disease in early stages of progression, monitoring treatment, and identifying patients at high risk of severe infection and death should be sought. Such biomarkers would enable clinicians do develop more detailed diagnostic protocols, more effective treatments and measures to prevent infection in patients at the highest risk of severe COVID-19.

This literature review summarizes the current knowledge on the diagnostic utility of chemokines as tools for monitoring the progression of COVID-19 and the health status of patients infected with SARS-CoV-2. Chemokine concentrations as inflammatory biomarkers could be used to more rapidly identify patients at high risk of severe infection and death.

## Systematic review

**Figure 1:**
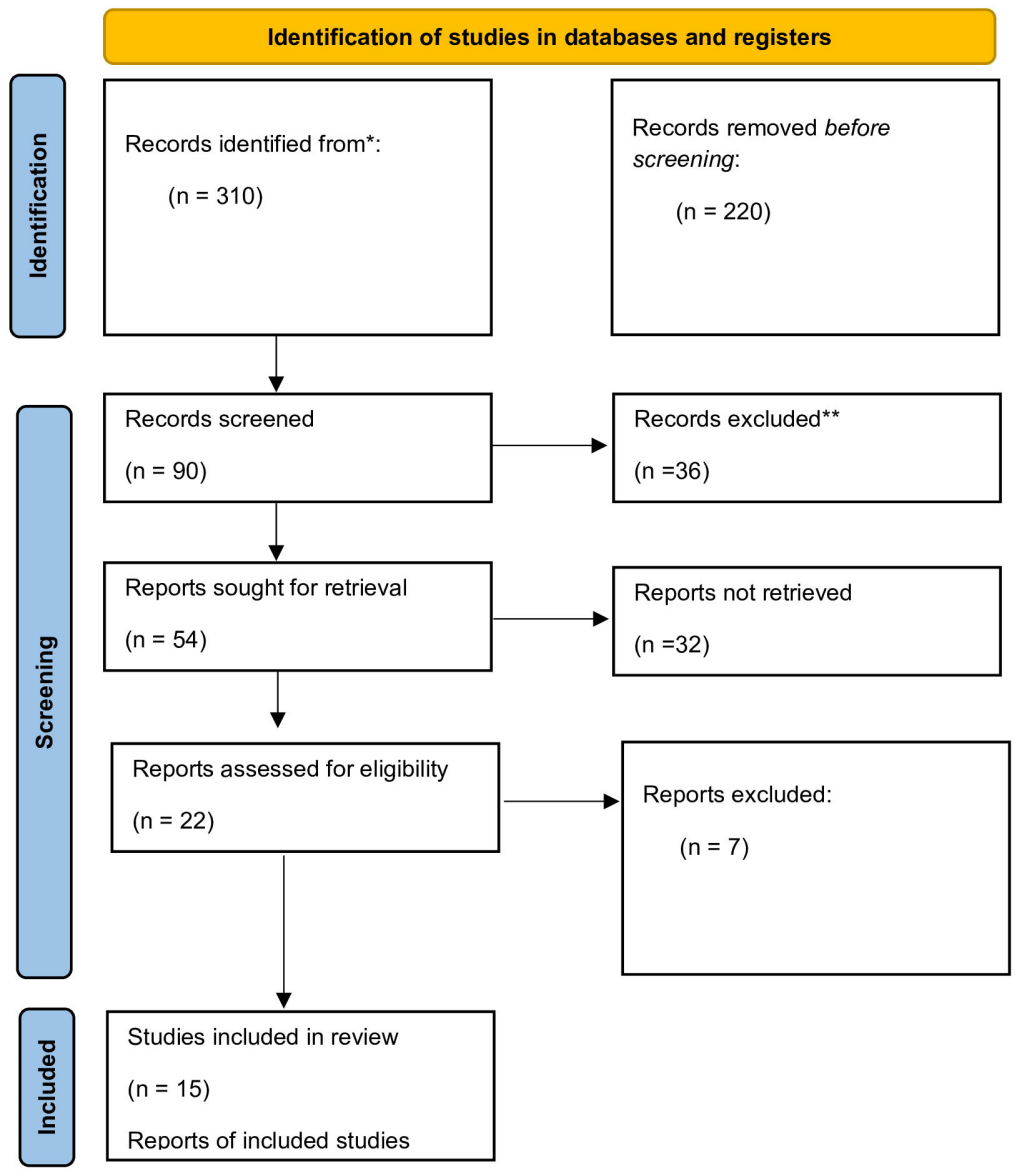
Flowchart of the systematic review process (Prisma).

The literature review was conducted in the PubMed database, and it involved articles published between 2020 and January 2024. The following keywords were used: COVID-19 and chemokines, SARS-CoV-2 virus and chemokines, COVID-19 and CCl2, COVID-19 and CCl3, COVID-19 and CCl5, COVID-19 and CXCl8, COVID-19 and CXCl10. The inclusion and exclusion criteria are presented in **Table 1**. The systematic review yielded 310 research articles in the Medline database (PubMed). Of those, 220 were rejected due to misleading titles. 90 abstracts were read, 22 were assessed for eligibility based on the adopted inclusion/exclusion criteria. 7 articles were unrelated to the studied topic. Ultimately, only 15 research articles were included in the study (**Figure 1**). The search and selection process are according to the PRISMA (16).

**Table 1:** Inclusion and exclusion criteria in the systematic review.

Inclusion criteria	Exclusion criteria
Articles in English	Articles in other languages
Articles describing the role of chemokines in COVID-19 (material: serum/plasma/CSF)	Articles not describing the role of chemokines in COVID-19 or the level of chemokines in other materials than serum/plasma/CSF
Research articles (*in vitro*, ex vivo, *in vivo*, clinical studies), meta-analyses, systematic literature reviews	Case studies, abstracts

## Structure of SARS-CoV-2

**Figure 2:**
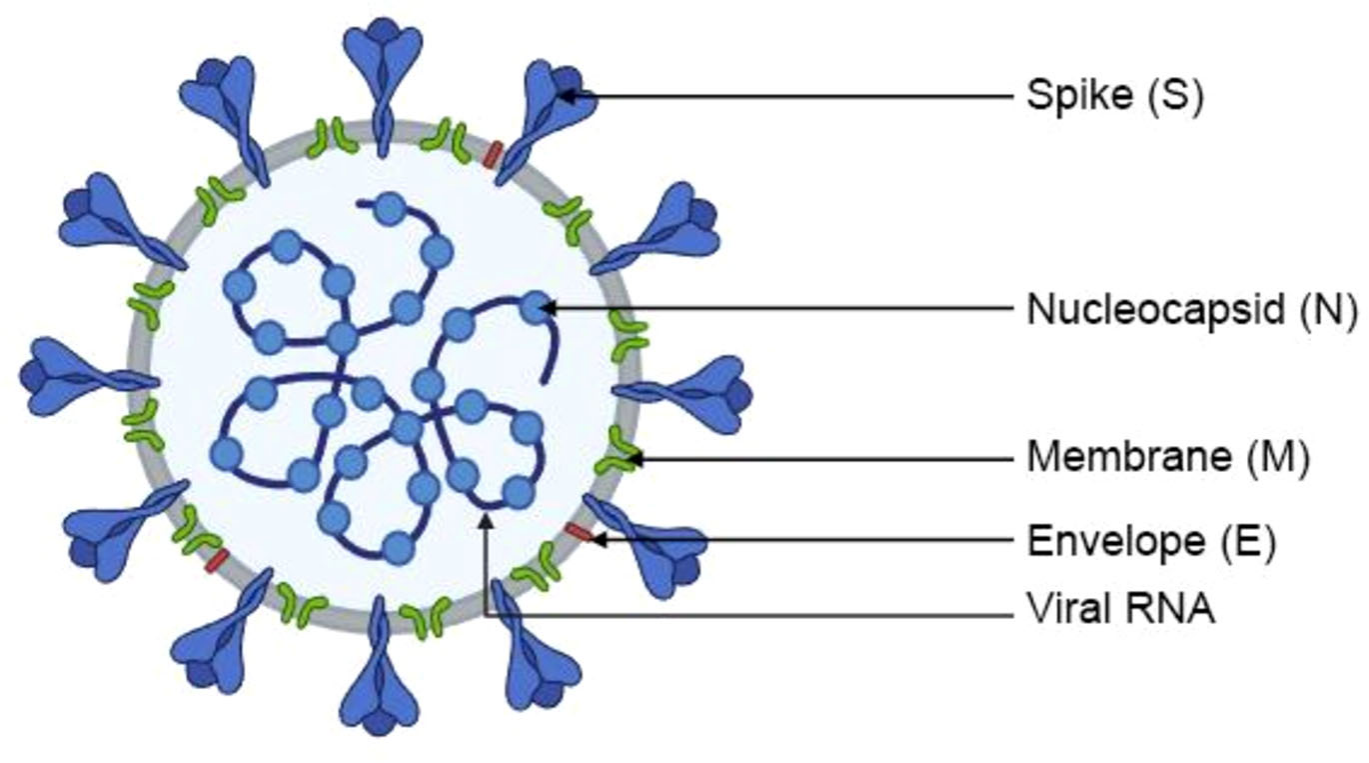
Structure of SARS-CoV-2.

Severe Acute Respiratory Syndrome Coronavirus 2 (SARS-CoV-2) belongs to a large and diverse family of Coronaviruses (*Coronaviridae*, CoV), which cause infections in animals - birds and mammals, including humans. SARS-CoV-2 is a β-coronavirus which, similarly to SARS-CoV and the Middle East Respiratory Syndrome Coronavirus (MERS-CoV), infects mammals. It is currently regarded as the most pathogenic virus in this group (17). SARS-CoV-2 and other Coronaviruses are spherical or oval particles with a lipid envelope containing proteins that enable the virus to penetrate host cells. Viral particles have a diameter of around 80–120 nm (18). The members of the Coronavirus family owe their name to the presence of spiculated surface glycoproteins that resemble a solar corona (19). SARS-CoV-2 is an enveloped, positive-sense ssRNA virus with a genome of ~29.9 kb. Its genetic material is composed of a positive-sense single-stranded RNA (ssRNA (+)) with an estimated length of 60–140 nm. The RNA contains around 30,000 nucleotides (20). The nucleotides are arranged into 14 open reading frames (ORFs) that represent two-thirds of information encoded within the virus’ genetic material and are responsible for the production of 16 non-structural proteins (NSPs). NSPs participate in the replication and transcription of new RNA molecules (21). The remaining one third of the genome encodes four major structural proteins (**Figure 2**) that play important roles in the pathogenesis of COVID-19, as well as accessory proteins that participate in virion-host cell interactions (22). The four major structural proteins that bind to host cells and elicit an immune response are the spike (S) protein - mediates receptor binding and membrane fusion, membrane (M) protein - shapes the envelope and assembly, envelope (E) protein - viroporin involved in assembly/release, and nucleocapsid (N) protein - packages the RNA genome (21). The S protein is a type I transmembrane glycoprotein that enables the virus to bind to cell membrane receptors and penetrate host cells. The S protein is composed of two functional subunits, S1 and S2. Subunit S1 binds to receptors on the surface of host cells, whereas S2 mediates the fusion of the viral envelope and the host cell membrane (**Figure 1**) (21). In mature SARS-CoV-2 particles, the S protein is assembled as a trimer with three binding domains that attach to the angiotensin-converting enzyme (ACE2) (23). Cryogenic electron tomography revealed the presence of around 30–40 trimeric S proteins on the surface of each viral particle in a pre-fusion state (24). The M protein is responsible for the shape of the viral envelope. Together with S and N proteins, the M protein interacts with the endoplasmic reticulum and the Golgi apparatus, where newly created virions are folded and released (25). The E protein is a viroporin, a small glycoprotein that speeds up the infectious process in the host organism. Protein-lipid pores are formed in the membranes of infected cells, and they can act as ion channels that increase membrane permeability and promote the release of progeny virions (26). The N protein binds, protects, and packages viral RNA to form ribonucleoprotein structures enclosed within the viral capsid (11).

## Cell entry mechanism and life cycle of SARS-CoV-2

**Figure 3:**
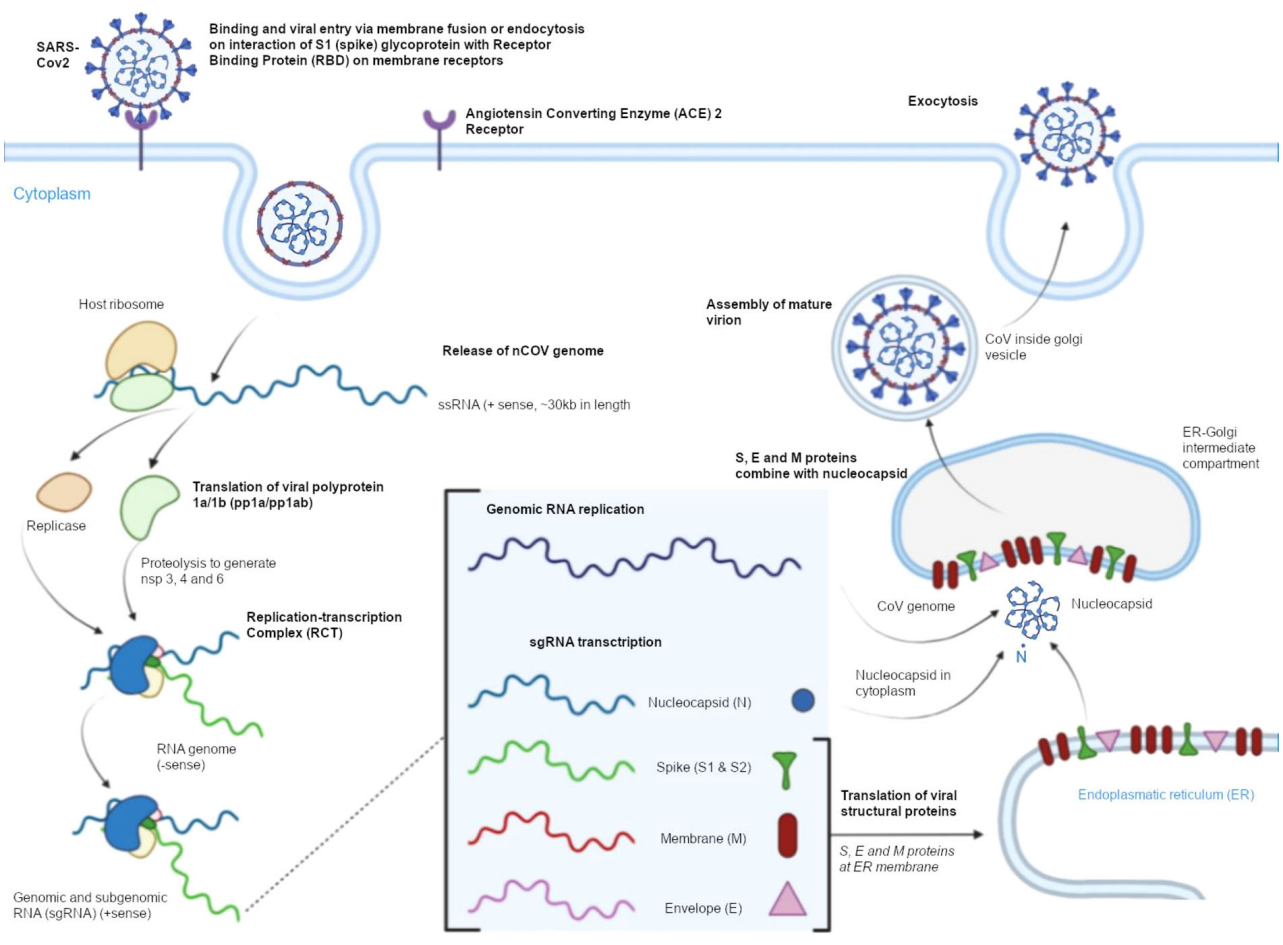
Cell entry mechanism and the life cycle of SARS-CoV-2.

SARS-CoV-2 easily enters host cells, which contributes to its high infectivity and is a key element in the pathogenesis of COVID-19 (**Figure 3**) (25). The pathogen uses human ACE2 as the entry receptor. ACE2 catalyzes the conversion of angiotensin II into angiotensin 1–7 which has vasodilating, anti-inflammatory, anti-thrombotic, and anti-proliferative properties. Under physiological conditions, ACE2 is expressed on type I and type II pneumocytes (26). According to research, the binding affinity of SARS-CoV-2 to ACE2 is around 10–20 stronger in comparison with other coronaviruses. The ACE2 receptor is localized on epithelial cells in the upper respiratory tract and lungs, enterocytes, gallbladder and bile duct cells, renal tubule cells, skeletal muscle cells, myocardial cells, and vascular endothelial cells (27). The S protein undergoes conformational changes when it binds to the ACE2 receptor, which increases its sensitivity to the host’s proteolytic enzymes (28). In the first stage of viral entry into host cells, the S protein is cleaved into subunits S1 and S2, and the proteolytic cleavage process is mediated by enzymes such as transmembrane protease serine 2 (TMPRSS2) and 4 (TMPRSS4), cathepsin L, trypsin, furin, and human airway trypsin-like protease. The infectivity of SARS- CoV-2 is determined by the S protein’s ability to bind with ACE2 and undergo cleavage because these processes promote endocytosis and facilitate the fusion of viral and endosomal membranes. This mechanism enables the virion to enter the host cell. The pH inside endosomes is low, which additionally promotes the fusion of the viral envelope with the endosomal membrane (29).

In the following stage, viral RNA is released and directly incorporated into host cell ribosomes. The SARS-CoV-2 replicase is translated, the virus’ genetic information is transcribed and replicated, and structural proteins are translated. In the final stage, new virions are folded in the endoplasmic reticulum – Golgi intermediate compartment (ERGIC). Newly formed, mature SARS-CoV-2 particles are transported by Golgi-derived vesicles to the plasma membrane and released from the infected cell into extracellular space in a process known as exocytosis (30–32).

## Inflammation caused by SARS-CoV-2

After entering host cells, viruses induce an inflammatory response by activating the immune system which is responsible for protecting the host organism and eliminating the pathogen. In viral infections, the innate (nonspecific) immune response is the first line of defense, which does not require prior contact with the pathogen (2).

Pattern/pathogen recognition receptors (PRRs) detect viruses in the human body. These receptors are found on the surface of cells or in the cytoplasm. PRRs are molecular alarms that sense pathogens in the host organism. They are present on the cells of the innate immune system, including neutrophils, monocytes, macrophages, dendritic cells, natural killer (NK) cells, endothelial cells, epithelial cells, and keratinocytes (33). These receptors recognize unknown viral protein patterns, activate immune cells, and trigger the release of pro- and anti- inflammatory cytokines (including IL-1, IL-6, IL-7, and TNF-α) and chemokines (including IP- 10). As a result, immune cells are rapidly recruited to the site of infection (3). PRRs have been divided into two types: receptors that are associated with the cell membrane, including toll-like receptors (TLRs) and C-type lectin receptors (CLRs), and cytoplasmic receptors, including NOD-like receptors (NLRs) and RIG-I-like receptors (RLRs) (34). The viral elements recognized by PRRs are known as pathogen-associated molecular patterns (PAMPs) which belong to a family of evolutionary conserved structural elements that are pathogen-specific and essential for the survival of pathogens. SARS-CoV-2 produces PAMPs such as ssRNA (detected by intracellular RLRs), the S protein and other envelope proteins (detected by TLRs). When SARS-CoV-2 is sensed, PRRs secrete interferons (IFNs), antiviral cytokines that play a key role in the immune response to viral infections. Type I-III IFNs participate in the primary (innate) immune response (35, 36). This group of cytokines includes IFN-α and IFN-β. Type I IFN binds to interferon-α/β receptors (INFAR) that are present on the surface of most cells. Transcription factors such as signal transducer and activator of transcription 1 (STAT1) are phosphorylated, which leads to the activation of interferon-stimulated genes (ISGs). ISGs participate in the inflammatory response and inhibit viral replication and transmission by slowing down cell metabolism, enhancing the secretion of pro-inflammatory cytokines, and increasing PRR expression (37, 38).

Viral entry and the resulting damage to host cells leads to the release of damage- associated molecular patterns (DAMPs) which are also recognized by PRRs. Adenosine triphosphate (ATP) and nucleic acids are the best-known DAMPs. Alarmins regulate the migration of macrophages, monocytes, and T cells to the site of infection and intensify the local inflammatory response by releasing pro-inflammatory cytokines. Excessive recruitment of immune cells causes tissue damage. In patients with severe COVID-19, the above can lead to lung inflammation observed in chest X-rays (39, 40).

SARS-CoV-2 has defense mechanisms, which prevent immune cells from sensing the virus and disrupt the host immune response. As a result, the virus can replicate and infect successive cells. Research has shown that SARS-CoV-2 inhibits IFN release, and in patients with severe COVID-19, IFN levels are lower than in patients with mild or moderate symptoms of the disease. SARS-CoV-2 also inhibits other antiviral innate immune responses by influencing PRRs and their signaling pathways (41, 42).

## Cytokine storm

**Figure 4:**
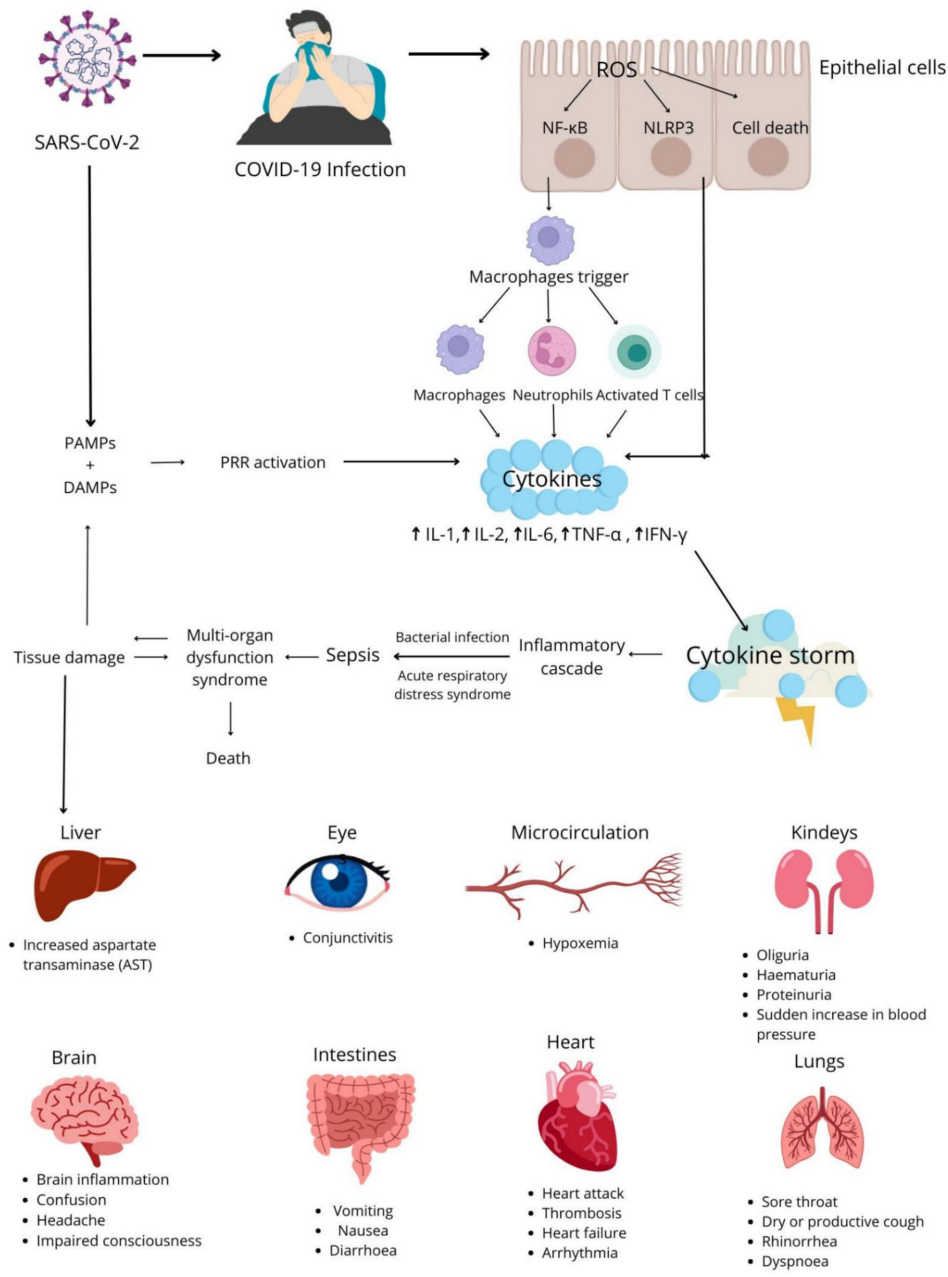
Pathomechanism of the cytokine storm and its consequences in COVID-19 patients.

Patients with SARS-CoV-2 infection can experience a wide range of clinical manifestations. Some infections present with mild symptoms or no symptoms and do not require treatment. However, severe infections can be life-threatening and may require hospitalization (43). Severe symptoms of COVID-19 are caused by both local and systemic inflammatory responses and the overproduction of pro-inflammatory factors, a process that is known as the cytokine storm or the cytokine release syndrome (CRS) (44). The cytokine storm is caused by the uncontrolled activation and enhancement of the host’s immune system (45), which leads to the massive release of cytokines and chemokines (46).Neutrophils, macrophages, NK cells, Th1, Th2, Th9 and Th12 lymphocytes, cytotoxic lymphocytes (Tc) (47), and cytokines, including IL-1, IL-6, IL-12, IL-18, TNF and IFN-γ are implicated in the pathogenesis of the cytokine storm (48, 49). Neutrophils promote clotting and produce cytokines. Macrophages present antigens, participate in phagocytosis, and induce cytokine production, whereas NK cells kill target cells and participate in immunoregulatory processes. In turn, IL-1 and IFN-γ produced by Th1 lymphocytes recruit macrophages and dendritic cells, and IL-4, IL-5 and IL-13 secreted by Th2 lymphocytes recruit eosinophils and basophils. IL-9 and IL-1 produced by Th9 lymphocytes activate mast cells, whereas IL-17, IL- 21 and IL-22 secreted by Th17 lymphocytes regulate neutrophil production. Tc lymphocytes produce IFN-γ, perforins and granzymes that exert cytotoxic effects on cells infected with SARS-CoV-2 (50, 51). IFN-γ and macrophages trigger a strong inflammatory response which leads to the cytokine storm. IFN-γ, IL-1, IL-6, IL-18, and TNF play the key role during CRS and trigger symptoms such as fever, headache, dizziness, and fatigue. The activation of the complement system is regulated by cytokines, which can lead to an increase or a decrease in cytokine production. The clinical symptoms of CRS include high fever, lymphadenopathy, hepatosplenomegaly, cytopenia, hyperferritinemia, and disorders of the central nervous system (CNS) (**Figure 4**) (52).

Chemokines, in particular monocyte chemoattractant protein-1 (MCP-1/CCL2), macrophage inflammatory protein-1α (MIP-1α/CCL3), MIP-1β/CCL4, the regulated on activation, normal T-cell expressed and secreted (RANTES/CCL5) chemokine, monokine induced by γ-interferon (MIG/CXCL9), and IFN-gamma-inducible protein (IP-10/CXCL10), also play important roles in COVID-19 (53, 54). These proteins are secreted by monocytes, macrophages, dendritic cells, blood platelets, fibroblasts, granulocytes, and epithelial cells in response to the identified pathogens, including SARS-CoV-2 (55). Chemokines are expressed in inflamed tissues, and they influence the maturation and differentiation of immune cells, including T cells, neutrophils, eosinophils, and macrophages. They also stimulate the migration of immune cells to the inflammation site to prevent acute respiratory failure (56). Chemokines fight inflammation and participate in tissue healing in COVID-19 patients (57).

Potential complications of COVID-19 include pneumonia and, in severe cases, ARDS, which decreases blood oxygen levels and leads to multiorgan failure and septic shock. ARDS, caused among others by the cytokine storm, is one of the most common causes of death in COVID-19 patients (27, 58). Enhanced inflammatory responses in COVID-19 can also cause damage to many other organs and tissues. The substances released during the cytokine storm are filtered by renal tubules, which can lead to acute kidney injury (AKI) (59). In COVID-19 patients, CRS also contributes to liver and bile duct fibrosis, and it can cause damage to the nervous system by increasing the risk of encephalopathy and stroke. Research studies examining the impact of SARS-CoV-2 on the CNS produced ambiguous results. It is assumed that the virus can cause hypoxemia, the cytokine storm can exert an indirect effect of on the brain, or the virus can directly damage the brain provided that it is able to cross the blood-brain barrier (60).

## Chemokines

Chemokines, or chemotactic cytokines, are a large group of proteins that are structurally homologous to cytokines, stimulate the movement of leukocytes and control their migration from the bloodstream to tissues. Chemokines participate in inflammatory processes and in the pathogenesis of many diseases, including COVID-19 (61). These polypeptides are composed of 66 to 111 amino acids with a tertiary structure stabilized by disulfide bonds between cysteines (62). Chemokines are functionally divided into constitutive chemokines and inducible chemokines that are formed under inflammatory conditions. Constitutive chemokines are produced in lymphoid tissues, including the bone marrow and the thymus, and they control basic migration processes and the development of immune system cells. Pro-inflammatory chemokines are secreted by various tissues and leukocytes in response to toxins and pro- inflammatory cytokines IL-1, TNFα, and IFNγ (63, 64). The chemokines described in this article belong to the group of inducible chemokines, and changes in their concentrations play an important role in different stages of SARS-CoV-2 infection (**Supplementary Table 1**).

## CCL2 (monocyte chemoattractant protein-1 – MCP1)

CCL2 is a small chemokine of the CC chemokine family. It is encoded by a gene on chromosome 17q12 (65). CCL2 recruits monocytes and basophils to the site of infection. It stimulates basophils and mast cells to release granules into extracellular space. This chemokine regulates the anti-cancer activity of monocytes and participates in the formation of granulomas (66, 67). CCL2 also recruits mast cells in the progenitor stage and increases neutrophil accumulation. This chemokine stimulates fibroblasts to produce procollagen (68).

During SARS-CoV-2 infection, CCL2 is overproduced by macrophage-derived vesicles, T cells, and lung endothelial cells. Monocytes, the main immune system cells, are activated, and they infiltrate lungs and elicit a strong inflammatory response. Acute inflammation damages the lung endothelium and may lead to diffuse alveolar hemorrhage and fibroproliferative disorders in patients with ARDS. Increased expression of CCL2 is associated with an acute inflammatory response in early stages of infection, and with prolonged stay in the ICU (53).

## CCL3 (macrophage inflammatory protein-1 alpha – MIP-1α)

CCL3, a chemokine of the CC family, is encoded by the CCL3 gene on chromosome 17q12 (69). This chemokine is secreted by macrophages and monocytes in response to the stimulation with bacterial endotoxin, as well as by the pro-inflammatory cytokine IL-1β. CCL3 can be also expressed by most hematopoietic cells, fibroblasts, endothelial cells, vascular smooth muscle cells, and activated blood platelets. This chemokine also acts as a ligand for receptors CCR1, CCR4, and CCR5 (70).

CCL3 contributes to inflammation through chemotaxis by directing the migration of inflammatory cells to tissues. It affects mainly monocytes, T cells, dendrocytes, NK cells, and blood platelets, and it activates granulocytes (65). This chemokine also induces the synthesis and release of other pro-inflammatory cytokines, including IL-1, IL-6, and TNF-α, from fibroblasts and macrophages (70).

Due to its functional properties, CCL3 plays an important role in viral infections, including COVID-19. A rapid and significant increase in CCL3 expression was observed in the blood serum of patients infected with SARS-CoV-2. The concentration of this chemokine was found to be higher in patients with moderate or severe COVID-19 than in asymptomatic patients, which suggests that CCL3 could be a useful biomarker for monitoring patients infected with SARS-CoV-2. It should also be noted that the pro-inflammatory effects of CCL3 could contribute to various pathological processes. This chemokine is implicated in the recruitment and activation/degranulation of granulocytes (in particular eosinophils), which can lead to an acute neutrophil-mediated inflammatory response and severe tissue damage (71).

## CCL5 (RANTES)

CCL5 is a pro-inflammatory chemokine of the CC family, and it is encoded by the CCL5 gene localized on chromosome 17q12 (72). CCL5 is chemotactic for T cells, eosinophils, basophils, monocytes, NK cells, dendrocytes, and mast cells (73, 74). Under the influence of IL-2 and IFN-γ secreted by T cells, CCL5 induces the proliferation and activation of NK cells known as CC-Chemokine-activated killer (CHAK) cells. This chemokine also induces the expression of extracellular matrix metalloproteinases (MMPs) which regulate the migration of immune cells to the site of inflammation (75). CCL5 is expressed late (3–5 days) after T cell activation via TCR, and this unusual kinetic profile is important for the maintenance of inflammation.

The role of CCL5 in COVID-19 has not been fully elucidated to date, and research studies analyzing this chemokine have produced contradictory results. It is generally believed that CD8+ T cells specific for SARS-CoV-2 are the main source of CCL5 in early stages of infection. In addition, the CCL5/CCR5 axis inhibits the apoptosis of macrophages that play a key role in the pathophysiology of COVID-19. However, elevated levels of CCL5 can also cause acute kidney and liver failure. These contradictory findings could be attributed to differences in the study populations and the duration and methods of measurement. Therefore, CCL5 cannot be univocally regarded as a reliable prognostic marker in COVID-19, and further research is needed to elucidate its role in SARS-CoV-2 infection (54).

## CXCL8 (interleukin 8 – IL-8)

CXCL8 is a pro-inflammatory chemokine which, similarly to other chemokines of the CXC family, is encoded by the CXCL8 gene on chromosome 4q13.3 (76). Macrophages and monocytes are the main sources of CXCL8, but this chemokine is also produced by other cells with toll-like receptors that participate in the innate immune response, as well as epithelial cells, airway smooth muscle cells, and endothelial cells. CXCL8 secretion is induced by factors IL- 17A and IL-17F which are produced by IL-6 and Th17 cells and whose levels are elevated in the peripheral blood of patients with SARS-CoV-2 infection (54). This chemokine binds mainly to receptors CXCR1 and CXCR2, and it shows higher affinity for CXCR1.

CXCL8 is implicated in the recruitment, activation, and accumulation of neutrophils. It induces chemotaxis, mainly in neutrophils, but also in other granulocytes. This chemokine also stimulates phagocytosis in the recruited cells. CXCL8 induces various physiological processes that stimulate adhesion to endothelial cells and migration to target tissues, including histamine release, increased expression of LFA-1 integrins on leukocyte membranes, increased concentration of intracellular Ca2+ ions, and the respiratory burst, a process in which reactive oxygen species (ROS) and digestive enzymes are activated to facilitate leukocyte migration to tissues (77). The activity of CXCL8 is strictly dependent on transcription factor AP-1 and associated with SARS-CoV-2 spike and nucleocapsid proteins. CXCL8 directly inhibits IFN induction by viral proteins, thus minimizing the anti-viral activity of IFN, in particular in early stages of infection (78). This chemokine also induces the production of neutrophil extracellular traps (NETs), strongly immunogenic and toxic networks of fibers that induce an inflammatory response and damage epithelial and endothelial cells. CXCL8 achieves these effects by stimulating exocytosis and the respiratory burst in neutrophils. In turn, NETs enhance the release of CXCL8, which increases the recruitment of neutrophils and prevents their apoptosis (54). Significantly elevated levels of CXCL8 were noted in patients who died from COVID-19 complications, which suggests that this chemokine can be used as a prognostic marker of mortality in patients infected with SARS-CoV-2 (51). IL-8 levels were found to be significantly higher in COVID-19 patients than in healthy subjects. In SARS-CoV-2 infection, uncontrolled production of IL-8, IL-2, IL-6, and TNF-α leads to the cytokine storm, disrupts immune system activity, and causes acute lung injury, ARDS (79–81), and multiorgan failure. Research has demonstrated that the risk of death from COVID-19 complications was positively correlated with an increase in IL-8 levels (82).

## CXCL10 (interferon gamma-induced protein 10 – IP-10)

CXCL10, a chemokine of the CXC family, is encoded by the CXCL10 gene on chromosome 4q.21.1 It is secreted by various cells, including monocytes, endothelial cells, and fibroblasts, in response to interferon gamma (83–85). CXCL10 binds mainly to receptor CXCR3 (86–89). CXCL10 participates in the recruitment of monocytes, macrophages, T cells, NK cells, and dendritic cells, and increases T cell adhesion to endothelial cells. CXCL10 modulates anti- cancer immune responses and promotes the inhibition of angiogenesis (90).

CXC10L is the key chemokine that participates in the TRL4-TRIF signaling pathway which is implicated in the pathogenesis of lung injury during viral infections. This chemokine contributes to the apoptosis of T cells and, consequently, lymphopenia in SARS-CoV-2 infection. Despite the fact that CXCL10 lacks the ELR sequence, it plays an important role in pulmonary neutrophil infiltration, which intensifies its production. The CXCL10/CXCR3 axis affects the recruited neutrophils and causes the respiratory burst, which contributes to inflammation and ARDS. CXCL10 is a reliable prognostic marker for risk assessment in patients infected with SARS-CoV-2 (86, 87).

## Conclusions

This literature review indicates that the immune response elicited by SARS-CoV-2 leads to the activation of various chemokines. The observed changes in chemokine levels are associated mainly with severe COVID-19, a poor prognosis, and a higher risk of mortality due to complications. The most recent research findings concerning chemokines’ role in the pathogenesis and progression of SARS-CoV-2 infection were summarized.

## Supplementary material

SUPPLEMENTARY TABLE 1 Review of the role of chemokines in COVID-19 infection.

## Glossary

ACE2: angiotensin-converting enzyme
AKI: Acute kidney injury
ARDS: Acute respiratory distress syndrome
ATP: Adenosine triphosphate
CHAK: CC-Chemokine-activated killer
CLRs: C-type lectin receptors
CNS: Central nervous system
CoV: Coronaviruses (*Coronaviridae*)
CRS: Cytokine release syndrome
CSF: Cerebral spine fluid
DAMPs: Damage-associated molecular patterns
ERGIC: Endoplasmic reticulum – Golgi intermediate compartment
G -CSF: granulocyte colony-stimulating factor
GM-CSF: granulocyte-macrophage colony-stimulating factor
ICU: Intensive care unit
IFN-γ: Interferon gamma
IFNs: Interferons
IL-1: Interleukin 1
IL-2: Interleukin 2
IL-4: Interleukin 4
IL-5: Interleukin 5
IL-6: interleukin 6
IL-7: interleukin 7
IL-9: interleukin 9
IL-10: Interleukin 10
IL-12: Interleukin 12
IL-13: Interleukin 13
IL-17: Interleukin 17
IL-17A: Interleukin 17A
IL-17F: Interleukin 17F
IL-18: Interleukin 18
IL-21: Interleukin 21
IL-22: Interleukin 22
INFAR: Interferon-α/β receptors
IP-10: Interferon gamma-induced protein 10, CXCL10
ISGs: Interferon-stimulated genes
M-CSF: Macrophage colony-stimulating factor
MCP-1: Monocyte chemoattractant protein-1, CCL2
MERS-CoV: Middle East Respiratory Syndrome Coronavirus
MIP-1α: Macrophage inflammatory protein-1α, CCL3
MIP-1β: Macrophage inflammatory protein-1β, CCL4
MMPs: Extracellular matrix metalloproteinases
NETs: Neutrophil extracellular traps
NK: Natural killer (cells)
NLRs: NOD-like receptors
NSPs: Non-structural proteins
ORFs: Open reading frames
PAMPs: Pathogen-associated molecular patterns
PRRs: Pattern/pathogen Pecognition receptors
RF: Respiratory failure
RLRs: RIG-I-like receptors
RNA: Ribonucleid acid
ROS: Reactive oxygen species
SARS-CoV-2: Severe Acute Respiratory Syndrome Coronavirus 2
SOFA: Sequential organ failure assessment
ssRNA: Single-stranded RNA
STAT1: Signal transducer and activator of transcription 1
Tc: Cytotoxic lymphocytes
Th1: T helper1 lymphocyte
Th2: T helper2 lymphocyte
Th9: T helper9 lymphocyte
Th12: T helper12 lymphocyte
TLRs: Toll-like receptors
TMPRSS2: Transmembrane protease serine 2
TMPRSS4: Transmembrane protease serine 4
TNF-α: Tumor necrosis factor alpha
WBC: White blood counts
