# Supplementary material for: Chemokines as potential biomarkers for predicting the course of COVID-19 – a review of the literature
Source: Front Immunol. 2025 Sep 23;16:1662643. doi: 10.3389/fimmu.2025.1662643 (PMC12500739; doi:10.3389/fimmu.2025.1662643)
Supplement: SUPPLEMENTARY TABLE 1 [file Table1.docx]

| Authors | Chemoki ne | Study group | Control group | Study group  Median | Contro l group Media n | P-value | Observations |
| --- | --- | --- | --- | --- | --- | --- | --- |
| Eichhorn et al. 2023  [92] | CCL2 | N = 78  sepsis patients with suspected bloodstream infection and a sequential organ failure assessment (SOFA)  score > 2  N = 14 of the above patients who were diagnosed with COVID-19 | N = 25  healthy individual s  N = 64  sepsis patients without COVID- 19 | 98.24 pg / mL | 23.2  pg / mL  34.22  pg/mL | - | Sepsis patients who tested positive or negative for COVID-19  had higher plasma CCL2 levels compared to healthy individuals. Sepsis patients with COVID- 19 had increased CCL2 levels compared to patients who tested negative for COVID- 19. |
| Blot et al. 2020 [93] | CCL2 | N = 14 ARDS  patients with COVID-19 | N = 7  healthy individual s  N = 7 ARDS  patients without COVID- 19 | Approx. 1 000  pg/mL in plasma | < 500  pg/mL in plasma  <  1 000  pg/mL in plasma | P<0.05  p>0.05 | Plasma CCL2 levels were significantly elevated in COVID-19  patients with ARDS relative to the control group. |
| Venkatara man et al. 2021[94] | CCL2 | Children aged 12 months to 18 years  N = 33  children with severe COVID-19 | Seronegat ive children that tested negative for COVID-  19 in a PCR test | >100  pg/mL | >10  pg/mL | P=0.005 | Children with PIMS-TS and children with severe COVID-19  had elevated serum CCL2 levels relative to the control group. |
|  |  | N = 44  children with pediatric inflammator y  multisystem |  | <1000  pg/mL |  | P<0.0001 |  |

|  |  | syndrome temporally associated with SARS- CoV-2 (PIMS-TS). PIMS-TS  occurs as a late manifestatio n of the SARS-CoV-  2 infection  (after 3-6 weeks), and many children present with multi-organ dysfunction s. |  |  |  |  |  |
| --- | --- | --- | --- | --- | --- | --- | --- |
| Jørgensen et al. 2020  [95] | CCL2 | N = 34  patients with COVID-19  (mean age: 58 years) patients with respiratory failure (RF) | N=13  patients without RF | Approx. 25 pg / mL | > 10  pg/mL | 0.0054 | Plasma CCL2 levels were significantly higher in the RF group than in non-RF patients upon admission. |
| Vu et al. 2021[96] | CCL2 | N = 13  patients with COVID-19 | N = 6  healthy individual s | 17.51  pg/mL in nasal washes (one week after a positive result of the RT- PCR test) | > 10  pg/mL in nasal washe s | p<0.05 | CCL2 level in nasal washes remained elevated for approx. 7 days after the onset of symptoms. |
| Guartazaca  -Guerrero et al. 2021  [97] | CCL2 | N = 2  patients with cerebrovasc ular disease and COVID-19 | Reference values for chemokin e levels in CSF | 15 000  pg/mL in CSF in case 1  337.51  pg/mL in CSF in case 2 | 160  pg/mL | - | CCL2 levels in CSF  considerably exceeded the reference values. These chemokines mediate the activation and recruitment of neutrophils, monocytes, and macrophages; therefore, the authors concluded that  the |

|  |  |  |  |  |  |  | neuroinflamma tion associated with cerebrovascula r disease in both patients resulted from an exacerbated inflammatory response rather than direct CNS invasion by SARS- CoV-2. |
| --- | --- | --- | --- | --- | --- | --- | --- |
| Ruhl et al. 2021[98] | CCL2 | N = 25  intensive care unit (ICU)  patients and 17  convalescen ts (CONV) | N = 29  unexpose d individual s | ICU –  approx. 100 pg/mL | UE –  approx  . 10  pg/mL  CONV –  approx  . - 10  pg/mL | p < 0.001  p < 0.001 | Plasma CCL2 levels were significantly higher in ICU patients than in CONV and unexposed individuals. |
| Korobova et al. 2022  [99] | CCL2 | N = 56  patients infected with the original SARS-CoV-  2 (mean  age: 66 years)  N = 95  patients infected with the Alpha variant of COVID-19  (mean age: 72 years)  N = 78  patients infected with the Delta variant variant (mean age: 71 years)  N = 57  patients  infected with the | N = 51  healthy donors (mean age: 49 years) | Approx. 600 pg/mL in plasma  >400  pg/mL in plasma  <600  pg/mL in plasma  > 400  pg/mL in plasma | <200  pg/mL in plasma | p<0.0001  (original vs Omicron p  = 0.0158)  p<0.0001  p<0.0001  p<0.0001 | All SARS- COV-2  variants induced the production of CCL2. Plasma CCL2 levels were significantly higher in infected patients than in healthy donors. Plasma CCL2 levels were highest in patients infected with the original SARS-CoV-2. |

|  |  | Omicron variant (mean age: 69 years) |  |  |  |  |  |
| --- | --- | --- | --- | --- | --- | --- | --- |
| Wolszczak  -  Biedrzycka et al. 2023  [5] | CCL2 | N=100  patients with mild and moderate COVID-19 | N=50  healthy individual s without a history of COVID- 19 | 119.5  pg/mL | 81.28  pg/mL | 0.1089 | CCL2 levels were higher in infected patients than in healthy controls. |
| Espindola et al. 2021  [100] | CXCL8 | N=48,  patients with neurological symptoms associated with COVID-19  H -  headache  E -  encephalopa thy  IND -  inflammator y  neurological diseases | N=10  healthy donors | H-S < 10  pg/mL  H-CSF  approx. 10 pg/mL  E-S -  approx. 15 pg/mL  E-CSF >  20 pg/mL  IND-S <  10 pg/mL  IND- CSF  > 30  pg/mL | C-S < 5  pg/mL in serum  C-CSF  < 5  pg/mL | p<0.05  p<0.01  p<0.001 | Patients with inflammatory neurological diseases had elevated levels of  CXCL8 in CSF  compared to controls.  Patients with encephalopath y had increased concentration of CXCL8 in CSF.  Serum levels of CXCL8  were higher in patients with encephalopath y than in controls. |
| Del Valle et al. 2020  [101] | CXCL8 | N=1 959  hospitalized patients with COVID-19  Median age: 63 years | N=9  healthy donors  Median age: 60 years | 42.4  pg/mL | < 20  pg/mL | p < 0.0001 | Serum CXCL8 levels were significantly elevated in COVID-19  patients relative to healthy donors. |
| Eichhorn et al. 2023  [92] | CXCL8 | N = 78  sepsis patients with suspected bloodstream infection and a sequential organ failure assessment | N = 25  healthy individual s | 15 pg/mL | 2.76  pg/mL | - | Patients who tested positive and negative for COVID-19  had higher plasma levels of IL-8 than the control group. |

|  |  | (SOFA)  score > 2  N = 14 of the above patients diagnosed with COVID-19 | N = 64  sepsis patients without COVID- 19 |  | 15.44  pg/mL |  |  |
| --- | --- | --- | --- | --- | --- | --- | --- |
| Blot et al. 2020 [93] | CXCL8 | N = 14 ARDS  patients with COVID-19 | N = 7  healthy individual s  N = 7 ARDS  patients without COVID- 19 | Approx. 50 pg/mL in plasma | < 10  pg/mL in plasma  Appro  x. 50  pg/mL in plasma | P<0.05  p>0.05 | Plasma CXCL8 levels were significantly higher in ARDS patients infected and not infected with COVID- 19 than in the control group. |
| Ruhl et al. 2021 [98] | CXCL8 | N = 25  intensive care unit (ICU)  patients and 17  convalescen ts (CONV) | N = 29  unexpose d individual s | ICU > 20  ppg/mL | UE < 10  pg/mL  CONV  < 10  pg/mL | p < 0.001  p < 0.001 | Plasma CXCL8 levels were significantly higher in ICU patients than in CONV (p <  0.001) and unexposed individuals (p  < 0.001). |
| Vu et al. 2021 [96] | CXCL8 | N = 13  patients with COVID-19 | N = 6  healthy individual s | 256.6  pg/mL in nasal washes (one week after a positive result of the RT- PCR test) | > 100  pg/mL in nasal washe s | p<0.05 | CXCL8 levels in nasal washes remained elevated for around 7 days after the onset of symptoms. |
| Guartazaca  -Guerrero et al. 2021  [97] | CXCL8 | N = 2  patients with cerebrovasc ular disease and COVID-19 | Reference values for chemokin e levels in CSF | 62680.36  pg/mL in CSF in case 1  377,67  pg/mL in CSF in case 2 | 67.5-  96.4  pg/mL | - | The levels of all analyzed chemokines were elevated. In particular, IL-8 levels in CSF  considerably exceeded the reference values. These  chemokines mediate the |

|  |  |  |  |  |  |  | activation and recruitment of neutrophils, monocytes, and macrophages; therefore, the authors concluded that the neuroinflamma tion associated with cerebrovascula r disease in both patients resulted from an exacerbated inflammatory response rather than direct CNS invasion by SARS- CoV-2. |
| --- | --- | --- | --- | --- | --- | --- | --- |
| Korobova et al. 2022  [99] | CXCL8 | N = 56  patients infected with the original SARS-CoV-  2 (mean  age: 66 years)  N = 95  patients infected with the Alpha variant of COVID-19  (mean age: 72 years)  N = 78  patients infected with the Delta variant (mean age: 71 years)  N = 57  patients infected with the | N = 51  healthy donors (mean age: 49 years) | Approx. 20 pg/mL in plasma  Approx. 12 pg/mL  Approx. 8 pg/mL  Approx. 7 pg/mL | <5  pg/mL in plasma | p<0.0001  (original virus vs delta and omicron p<0.0001)  p<0.0001  p<0.0001  p<0.0001 | All SARS-  CoV-2 variants induced the production of CXCL8.  Plasma levels of CXCL8  were significantly higher in patients than in healthy donors. The original  SARS-CoV-2  induced the highest production of CXCL8. |

|  |  | Omicron variant (mean age: 69 years) |  |  |  |  |  |
| --- | --- | --- | --- | --- | --- | --- | --- |
| Bergantini  L. et al. 2022 [86] | CXCL8 | N= 64  patients with COVID-19 | N= 27  healthy individual s | 223.4  pg/mL | 64.8  pg/mL | p<0.0001 | CXCL8 is a good biomarker for differentiating between COVID-19  patients and healthy controls. |
| Cabaro S. et al. 2021  [102] | CXCL8 | N=19  patients with severe COVID-19 | N=49  healthy individual s | 171.5  pg/mL [63.86;  1122] | 16.09  pg/mL [13.42  ; 18.6] | p < 0.0001 | CXCL8 is a highly useful diagnostic and prognostic biomarker for classifying COVID-19  patients (AUC  > 0.95). It can be used to predict the severity of COVID-19  and make decisions in clinical practice. |
| Bülow Anderberg  S. et al. 2021 [103] | CXCL8 | N=24  patients with confirmed COVID-19 | - | 22.15  pg/mL | 5.02  pg/mL | P<0.05 | CXCL8 can be a useful biomarker for monitoring COVID-19  patients because it is correlated with the mortality rate. Serum levels of CXCL8 are correlated with the severity of  COVID-19. |
| Wolszczak  -  Biedrzycka et al. 2023  [5] | CXCL8 | N=100  patients with mild and moderate COVID-19 | N=50  healthy subjects without a history of COVID- 19 | 33.38  pg/mL | 9.11  pg/mL | < 0.0001 | CXCL8 is a highly effective biomarker for diagnosing COVID-19  patients. |
| Jørgensen et al. 2020  [95] | CCL5 | N = 34  patients with COVID-19 | N=13  patients without RF | Approx. 2  *10^3^  pg/mL | Appro  x. 2  *10^3^  pg/mL | - | Plasma CCL5 levels did not differ between  RF and non- RF patients |

|  |  | (mean age: 58 years)  N = 21  patients with respiratory failure (RF) |  |  |  |  | upon admission. |
| --- | --- | --- | --- | --- | --- | --- | --- |
| Blot et al. 2020 [93] | CCL5 | N = 14 ARDS  patients with COVID-19 | N = 7  healthy individual s  N = 7 ARDS  patients without COVID- 19 | < 50000  pg mL in plasma | < 50000  pg/mL in plasma  <2 500  pg/mL in plasma | p>0.05  p = 0.025 | Plasma CCL5 levels were significantly higher in ARDS patients with COVID- 19 than in ARDS patients without COVID-19. |
| Wang et al. 2021 [104] | CCL5 | N = 5  patients with severe COVID-19 | N = 24  healthy individual s | <250  pg/mL in patients with severe COVID-  19 and in convalesce nts | Appro  x. 300  pg/mL | P<0.05  P<0.05 | Serum CCL5 levels were lower in patients with severe COVID-19  and in convalescents than in the control group. |
| Venkatara man et al. 2021 [94] | CCL5 | Children aged 12 months to 18 years  N = 33  patients with severe COVID-19  N = 44  children with pediatric inflammator y multisystem syndrome temporally associated with SARS- CoV-2 (PIMS-TS). PIMS-TS  occurs as a late  manifestatio | Control group – seronegati ve children who tested negative for COVID-  19 in the PCR test | <10000  pg/mL  <10000  pg/mL  <10000  pg/mL | >1000  pg/mL | P<0.0001  P=0.0007  P<0.0001 | All patients with COVID- 19-related infections (children with PIMS-TS,  severe COVID-19,  and seropositive children) had higher serum CCL5 levels than healthy, seronegative children. |

|  |  | n of the SARS-CoV-  2 infection  (after 3-6 weeks), and many children present with multi-organ dysfunction s.  N=47  seropositive children without PIMS-TS |  |  |  |  |  |
| --- | --- | --- | --- | --- | --- | --- | --- |
| Eichhorn et al. 2023  [92] | CCL5 | N = 78  sepsis patients with suspected bloodstream infection and a sequential organ failure assessment (SOFA)  score > 2 | N = 25  healthy individual s | 5319 pg / mL | 7742  pg/mL | - | Patients who tested positive or negative for COVID-19  had lower plasma CCL5 levels than the control group. |
| Guartazaca  -Guerrero et al. 2021  [97] | CCL5 | N = 2  patients with cerebrovasc ular disease and COVID-19 | Reference values for chemokin e levels in CSF | 261.5  pg/mL in CSF in case 1  45.15  pg/mL in CSF in case 2 | 5.43 –  226.8  pg/mL | - | CCL5 levels were elevated (case 1) or within the reference limit (case 2). |
| Wolszczak  -  Biedrzycka et al. 2023  [5] | CCL5 | N=100  patients with mild and moderate COVID-19 | N=50  healthy subjects without a history of COVID- 19 | 11868  pg/mL | 16538  pg/mL | 0.0019 | CCL5 levels were higher in COVID-19  patients than in healthy controls. |
| Ruhl et al. 2021 [98] | CXCL10 | N = 25  intensive care unit (ICU)  patients and 17  convalescen ts (CONV) | N = 29  unexpose d individual s | ICU ->  1000 pg mL | UE –  approx  . 100  pg/mL  CONV  > 100  pg/mL | p < 0.001  p < 0.001 | Plasma CXCL10  levels were significantly higher in ICU patients than in CONV and unexposed individuals. |

| Espindola et al. 2021  [100] | CXCL10 | N=48  patients with neurological symptoms associated with COVID-19  H -  headache  E -  encephalopa thy  IND - | N=10  healthy donors | H-S >100  pg/mL  H-CSF  <300  pg/mL  E-S -  approx. 300 pg/mL  E-CSF <  300 pg/mL  IND-S >1  00 pg/mL  IND- CSF  > 1 000  pg/mL | C-S < 100  pg/mL  C-CSF  < 100  pg/mL | p<0.01 | Patients with inflammatory neurological diseases had higher levels of CXCL10 in CSF than controls. |
| --- | --- | --- | --- | --- | --- | --- | --- |
| Rizzi et al. 2022 [105] | CXCL10 | N = 139  hospitalized COVID-19  patients, 7 days after admission | - | > 4271 pg  /mL | - | p<0.001 | Seven days after admission, serum CXCL10  concentrations higher than 4271 pg/mL are indicative of clinical worsening. |
| Wang et al. 2021 [104] | CXCL10 | N = 5  patients with severe COVID-19  N= 16  patients with moderate COVID-19 | N = 24  healthy individual s | Approx. 200 pg/mL in serum in the acute (A) stage and 100 pg/mL in convalesce nt stage (C)  Approx. 100 pg/mL  (A) and 50 pg/mL (C) | <50  pg/mL | p<0.001  p>0.05 | CXCL10  levels were higher in patients with severe and moderate COVID-19 in  the acute and convalescent stages of the disease than in the control group. |
| Venkatara man et al. 2021 [94] | CXCL10 | Children aged 12 months to 18 years  N = 33 with severe COVID-19  N = 44  children with pediatric inflammator | Control group – seronegati ve children who tested negative for COVID-  19 in the PCR test | >100  pg/mL  >1000  pg/mL | Appro  x. 10  pg/mL | P<0.0001  P=0.0043 | All patients with COVID- 19-related infections (children with PIMS-TS,  severe COVID-19,  and seropositive children) had elevated serum CCL5 levels |

|  |  | y multisystem syndrome temporally associated with SARS- CoV-2 (PIMS-TS). PIMS-TS  occurs as a late manifestatio n of the SARS-CoV-  2 infection  (after 3-6 weeks), and many children present with multi-organ dysfunction s.  N=47  seropositive children without PIMS-TS |  | Approx. 100 pg/mL |  | P=0.009 | relative to healthy, seronegative children |
| --- | --- | --- | --- | --- | --- | --- | --- |
| Eichhorn et al. 2023  [92] | CXCL10 | N = 78  sepsis patients with suspected bloodstream infection and a sequential organ failure assessment (SOFA)  score > 2  N = 14 of the above patients diagnosed with COVID-19 | N = 25  healthy individual s  N = 64  sepsis patients without COVID- 19 | 5806  pg/mL | 297  pg/mL  846  pg/mL | - | Patients who tested positive and negative for COVID-19  had higher plasma levels of IL-8 than the control group. Plasma CXCL10  levels were higher in sepsis patients with COVID- 19 than in sepsis patients who tested negative for COVID-19. |
| Blot et al. 2020 [93] | CXCL10 | N = 14 ARDS  patients with COVID-10 | N = 7  healthy individual s | < 1 000  pg/mL in plasma | > 200  pg/mL in plasma | p<0.05 | Plasma CXCL10  levels were significantly higher in ARDS |

|  |  |  | N = 7 ARDS  patients without COVID- 19 |  | > 500  pg/mL in plasma | p>0.05 | patients with COVID-19  compared to healthy controls, but not in ARDS patients without  COVID-19. |
| --- | --- | --- | --- | --- | --- | --- | --- |
| Jørgensen et al. 2020  [95] | CXCL10 | N = 34  patients with COVID-19  (mean age: 58 years) and with respiratory failure (RF) | N=13  patients without RF | Approx. 2  *10^3^  pg/mL | > 1 *  10^3^  pg/mL | P=0.039 | Plasma CXCL10  levels were significantly higher  in the RF group than in non-RF patients upon admission. |
| Lore et al. 2021 [106] | CXCL10 | N = 111  patients with COVID-19 | - | > 4782  pg/mL  >16663  pg/mL | -  - | -  p=0.0002 | A CXCL10  value of 4782 pg/mL was the cut-off point for patient stratification based on the risk of transfer to ICU. 27 out of 51 patients with CXCL10  > 4784 pg/mL were transferred to the ICU, while only 9 out of 60 were transferred when CXCL10 was below 4782 pg/mL.  A CXCL10  value of 16,633 pg/mL was the cut-off point for classifying patients with a low and high risk of death. 12 out of 19 patients with CXCL10 >  16,663 pg/mL died, while only 10 out of 92 died when CXCL10 was  below 16,663 pg/mL. |

| Vu et al. 2021 [96] | CXCL10 | N = 13  patients with COVID-19 | N = 6  healthy individual s | 214.4  pg/mL in nasal washes (one week after a positive result of the RT- PCR test) | > 50  pg/mL in nasal washe s | p<0.05 | CXCL10  levels in nasal washes remained elevated for 2-  3 weeks after the onset of symptoms (a significant increase was noted 7 days after the onset of symptoms). |
| --- | --- | --- | --- | --- | --- | --- | --- |
| Korobova et al. 2022  [99] | CXCL10 | N = 56  patients infected with the original SARS-CoV-  2 (mean  age: 66 years)  N = 95  patients infected with the Alpha variant of COVID-19  (mean age: 72 years)  N = 78  patients infected with the Delta variant (mean age: 71 years)  N = 57  patients infected with the Omicron variant (mean age: 69 years) | N = 51  healthy donors (mean age: 49 years) | > 10000  pg/mL in plasma  > 1000  pg/mL in plasma  > 10000  pg/mL in plasma  Approx. 10000  pg/mL in plasma | > 100  pg/mL in plasma | p<0.0001  p<0.0001  p<0.0001(D  elta vs Alpha p=0.0098vs Omicron p<0.001)  p<0.0001 | All SARS- COV-2  variants induced the production of CXCL10.  Plasma CXCL10  levels were significantly higher patients than in healthy donors. The Delta variant of COVID-19  induced the highest production of CXCL10. |
| Wolszczak  -  Biedrzycka et al. 2023  [5] | CXCL19 | N=100  patients with mild and moderate COVID-19 | N=50  healthy subjects without a history of | 1301  pg/mL | 93.09  pg/mL | p<0.0001 | CXCL10 could be an effective biomarker for differentiating between COVID-19 |

|  |  |  | COVID- 19 |  |  |  | patients and healthy subjects (AUC=0.9025)  . |
| --- | --- | --- | --- | --- | --- | --- | --- |
| Eichhorn et al. 2023  [92] | CCL3 | N = 78  sepsis patients with suspected bloodstream infection and a sequential organ failure assessment (SOFA)  score > 2  N = 14 of the above patients were diagnosed with COVID-19 | N = 25  healthy individual s  N = 64  sepsis patients without COVID- 19 | 3.2 pg/mL | 1.28  pg/mL  5.42  pg/mL | P<0.05 | Patients who tested positive and negative for COVID-19  had higher plasma levels of CCL3 than the control group. |
| Jørgensen et al. 2020  [95] | CCL3 | N = 34  patients with COVID-19  (mean age: 58 years) and with respiratory failure (RF) | N=13  patients without RF | Approx.  2.5 pg/mL | > 2  pg/mL | 0.034 | Plasma CCL3 levels were significantly higher in the RF group than in non-RF patients upon admission. |
| Guartazaca  -Guerrero et al. 2021  [97] | CCL3 | N = 2  patients with cerebrovasc ular disease and COVID-19 | Reference values for chemokin e levels in CSF | 58.06  pg/mL in CSF in case 1  10 pg/mL in CSF in case 2 | 4.69  pg/mL | - | CCL3 levels in CSF  significantly exceeded the reference values.  Considering the fact that viral mRNA was not detected in CSF, and that these chemokines mediate the activation and recruitment of neutrophils, monocytes and macrophages, the authors  concluded that the |

|  |  |  |  |  |  |  | neuroinflamma tion associated with cerebrovascula r disease in both patients resulted from an exacerbated inflammatory response rather than direct CNS invasion by SARS- CoV-2. |
| --- | --- | --- | --- | --- | --- | --- | --- |
| Korobova et al. 2022  [99] | CCL3 | N = 56  patients infected with the original SARS-CoV-  2 (mean  age: 66 years)  N = 95  patients infected with the Alpha variant of COVID-19  (mean age: 72 years)  N = 78  patients infected with the Delta variant (mean age: 71 years)  N = 57  patients infected with the Omicron variant (mean age: 69 years) | N = 51  healthy donors (mean age: 49 years) | >50 pg/mL in plasma  Approx. 50 pg/mL in plasma  Approx. 10 pg/mL in plasma  >10 pg/mL in plasma | Appro  x. 10  pg/mL in plasma | p<0.0001  (original vs Delta and Omicron p<0.0001)  p<0.0001  (Alpha vs Delta and Omicron p<0.0001)  p>0.05  p>0.05 | The original SARS-CoV-2  and the Alpha variant of COVID-19  induced the production of CCL3. Plasma CCL3 levels were significantly higher in these patients than in healthy donors. The original  SARS-CoV-2  induced the highest production of this chemokine. |
| Wolszczak  -  Biedrzycka et al. 2023  [5] | CCL3 | N=100 with mild and moderate COVID-19 | N=50  healthy subjects without a history of COVID- 19 | 3.33  pg/mL | 1.735  pg/mL | 0.0001 | CCL3 levels were significantly higher in COVID-19  patients than in healthy controls. |

| 5. | B. Wolszczak-Biedrzycka, Changes in chemokine and growth factor levels may be useful biomarkers for |
| --- | --- |
|  | monitoring disease severity in COVID-19 patients; a pilot study, Front Immunol (2024). 4:14:1320362. |
|  | doi: 10.3389/fimmu.2023.1320362. |
| 92. | T. Eichhorn, Infection with SARS-CoV-2 Is Associated with Elevated Levels of IP-10, MCP-1, and IL-13 in |
|  | Sepsis Patients, Diagnostics (Basel). (2023); 13(6): 1069. |
| 93. | M. Blot, CXCL10 could drive longer duration of mechanical ventilation during COVID-19 ARDS, Crit Care. |
|  | (2020); 24: 632. |
| 94. | A. Venkataraman, Plasma biomarker profiling of PIMS-TS, COVID-19 and SARS-CoV2 seropositive |
|  | children – a cross-sectional observational study from southern India, eBioMedicine. (2021); 66: |
|  | 103317. <https://doi.org/10.1016/j.ebiom.2021.103317> |
| 95. | M. Jørgensen, Increased interleukin-6 and macrophage chemoattractant protein-1 are associated with |
|  | respiratory failure in COVID-19, Scientific Reports (2020); 10: 21697 (2020) |
| 96. | D. Vu, Longitudinal Analysis of Inflammatory Response to SARS-CoV-2 in the Upper Respiratory Tract |
|  | Reveals an Association with Viral Load, Independent of Symptoms, Journal of Clinical Immunology |
|  | (2021); 41: 1723–173. https://doi.org/10.1007/s10875-021-01134-z |
| 97. | S. Guartazaca-Guerrero S, High Levels of IL-8 and MCP-1 in Cerebrospinal Fluid of COVID-19 Patients |
|  | with Cerebrovascular Disease. Exp Neurobiol (2021);30:256-261. <https://doi.org/10.5607/en21009> |
| 98. | L. Ruhl, Endothelial dysfunction contributes to severe COVID-19 in combination with dysregulated |
|  | lymphocyte responses and cytokine networks, Signal Transduction and Targeted Therapy volume |
|  | (2021); 418: <https://doi.org/10.1038/s41392-021-00819-6> |
| 99. | Z. Korobova, A Comparative Study of the Plasma Chemokine Profile in COVID-19 Patients Infected with |
|  | Different SARS-CoV-2 Variants, J Mol Sci; (2022) Aug 13;23(16):9058. doi: 10.3390/ijms23169058. |

| 100. | O.M. Espíndola, Inflammatory Cytokine Patterns Associated with Neurological Diseases in Coronavirus |
| --- | --- |
|  | Disease 2019, Ann Neurol. (2021) May; 89(5): 1041–1045. doi: 10.1002/ana.26041 |
| 101. | Diane Marie Del Valle, An inflammatory cytokine signature predicts COVID-19 severity and survival, |
|  | Nature Medicine; (2020); 26: 1636–1643. <https://doi.org/10.1038/s41591-020-1051-9> |
| 102. | S. Cabaro, Cytokine signature and COVID-19 prediction models in the two waves of pandemics, Sci |

(2021); 11: 20793, doi: 10.1038/s41598-021-00190-0.

103. S.B. Anderberg, Increased levels of plasma cytokines and correlations to organ failure and 30-day mortality in critically ill Covid-19 patients, Cytokine (2021); Feb:138:155389. doi: 10.1016/j.cyto.2020.155389. 104. Y. Wang, COVID-19: Inflammatory Profile, Annual Review of Medicine, (2022);73:65-80, 7d1o9i.org/10.1146/annurev-med-042220-012417

105. M. Rizzi, Prognostic Markers in Hospitalized COVID-19 Patients: The Role of IP-10 and C-Reactive protein

Disease Markers (2022); 2, <https://doi.org/10.1155/2022/3528312>

106. N. Lore, CXCL10 levels at hospital admission predict COVID-19 outcome: hierarchical assessment of 53 7p2u3tative inflammatory biomarkers in an observational study, Mol Med, (2021);27(1):129. doi: 71204.1186/s10020-021-00390-4.
